# Design of Ratio-Fluorescence Nanohybrid Based on Radix *Hedysari* Green-Synthesized CDs and GSH-AuNCs for Sensitive Detection of Cefodizime Sodium in Urine Sample

**DOI:** 10.3390/ijms25115971

**Published:** 2024-05-29

**Authors:** Yan-Xin Guo, Xin-Ran Guo, Xin-Yue Chen

**Affiliations:** Institute of Pharmaceutical Analysis, School of Pharmacy, Lanzhou University, Lanzhou 730030, China; gyanxin2023@lzu.edu.cn (Y.-X.G.); guoxr21@lzu.edu.cn (X.-R.G.)

**Keywords:** ratio fluorescence, green synthetic carbon quantum dots, gold nanoclusters, cefodizime sodium

## Abstract

A dual-emission ratio-fluorescent sensing nanohybrid based on Radix *Hedysari* green-synthesized carbon quantum dots (CDs) and glutathione-functionalized gold nanoclusters (GSH-AuNCs) had been developed for the determination of cefodizime sodium (CDZM). The designed fluorescence nanohybrid had two significant fluorescence emission peaks at 458 nm and 569 nm when excited at 360 nm, which was attributed to the CDs and GSH-AuNCs. With the addition of CDZM, the fluorescence at 458 nm was slightly weakened while the fluorescence at 569 nm was enhanced obviously. Based on the relationship between the I_569_/I_458_ fluorescence intensity ratio and the concentration of CDZM, the designed nanohybrid exhibited a good linearity range of 1.0–1000.0 μM and the limit of detection (LOD) was 0.19 μM. The method was finally applied in the detection of CDZM in urine, showing the potential applications in complicated biological samples.

## 1. Introduction

β-lactam antibiotics are frequently prescribed antimicrobial drugs including penicillin and cephalosporins. Cephalosporins, the drugs of choice for a large number of infectious diseases, are broadly used in many regions for both humans and animals owing to their potent antibacterial activity and capacity to penetrate the cytoderm [1,2]. However, the excessive accumulation of cephalosporins due to the improper and excessive use in daily life results in bacterial resistance and potential harm to human health [3]. Therefore, it is of great significance that we develop a new analytical method for the effective determination of cephalosporin. Several analytical methods have been reported for tracking cephalosporins in daily life, such as high-performance thin-layer chromatography densitometry [4], capillary liquid chromatography [5], liquid chromatography [6,7,8,9], and spectrophotometric methods [10]. These methods have a high sensitivity and good accuracy, but inevitably suffer from the disadvantages of expensive equipment, a complicated operation process, and an excessive use of organic solvents [11]. Thus, fluorescence detection appears and draws much attention, attributed to the advantages of a simple operation, visual detection, and fast analysis, which are beneficial for real-time portable detection. Among many fluorescence design strategies, the dual-wavelength ratio-fluorescence detection performs particularly well. It has the ability to avoid false-negative or false-positive results caused by the interference of environment, instrument, and other irrelevant factors due to its self-calibration effect [12,13,14,15].

Carbon dots (CDs) are zero-dimensional carbon nanomaterials with the advantages of tunable photoluminescence behavior, good fluorescence stability, low toxicity, and high biocompatibility [16,17], exhibiting the large potential in fluorescence sensing [18], photocatalysis [19], targeted delivery [20], biological imaging [21], and antimicrobial activity [22]. The chemical method is a common method with which to synthesize CDs because of its simple operation and high reliability. However, the overuse and improper handling of chemical agents can seriously harm the environment and public health. Fortunately, CDs can also be easily prepared using a large number of low-cost and readily available green precursors as raw materials, showing the obvious advantages of being natural, non-toxic, and renewable [23,24]. Radix *Hedysari*, a time-honored folk herb used in traditional China, has good pharmacological effects [25,26] attributed to the various components of flavonoids, triterpenoids and triterpenoid saponins, coumarins, organic acids, and polysaccharides [27,28]. In addition to their efficacy in diseases, these ingredients have also shown good biological activity, which would act as an excellent carbon source. There have been no studies reported on its use yet; thus, it is worth trying to use Radix *Hedysari* as a green precursor for synthetic CDs.

Gold nanoclusters (GSH-AuNCs) have undergone rapid progress in recent years as a result of their unique nano-properties and excellent optical performance, and they are widely used in sensing, biological imaging, and other fields [29]. Glutathione (GSH) is a tripeptide containing gamma-amide bonds and sulfhydryl groups with glutamic acid, cysteine, and glycine included. Considering its good reducibility and biological activity, it was always used to synthesize GSH-functionalized GSH-AuNCs (GSH-AuNCs) with active groups on the surface. This article designed a ratio-fluorescence nanohybrid in the basis of CDs and GSH-AuNCs for CDZM detection. In recent years, ratio-fluorescence probes based on CDs and AuNCs complexes have been widely studied for their anti-interference capability and high stability [30,31,32]. Zhang et al. [33] reported a nanohybrid ratio-fluorescence probe (AuNCs/CQDs) by simply mixing, realizing the Hg^2+^ detection in cosmetics and wastewater. Yang et al. [34] designed the nanoprobe mixture of cyan CDs and red-emitting AuNCs, with the aid of mercury ions (Hg^2+^), realizing the iodide ion detection based on an “on−off−on” sensing mechanism.

**Scheme 1:**
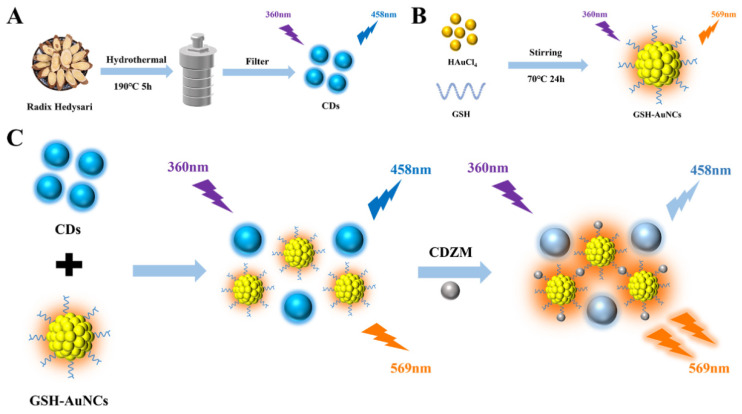
Schematic illustration of the synthesis of the CDs (**A**), GSH-AuNCs (**B**), the formation of the GSH-AuNCs/CDs nanohybrid and the sensing principle of detecting CDZM (**C**).

Herein, we first reported the green synthesis of CDs using the extract of Radix *Hedysari*. GSH-AuNCs were also well-prepared with a strong luminescence. A ratio-fluorescence probe was established by physically mixing the CDs and GSH-AuNCs, in which CDs emitted blue fluorescence and GSH-AuNCs emitted orange fluorescence. As showed in Scheme 1, when the excitation wavelength was 360 nm, the probe had two fluorescence emission peaks at 458 nm and 569 nm corresponding to two kinds of luminous nanomaterials. After the addition of CDZM, the fluorescence at 458 nm was weakened while the fluorescence at 569 nm was enhanced. A good linear equation was established between the concentration and fluorescence intensity ratio, realizing the highly sensitive and low detection limit of CDZM. In the end, the established fluorescence nanohybrid was successfully applied in the human urine sample.

## 2. Results and Discussion

### 2.1. Characterization of CDs, GSH-AuNCs, and GSH-AuNCs/CDs Nanohybrid

**Figure 1:**
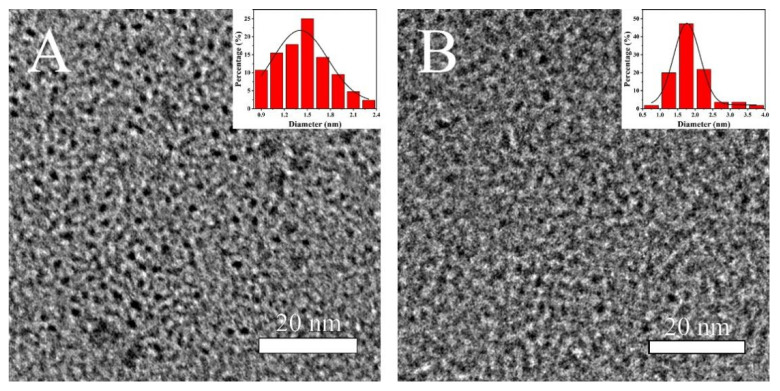
TEM images with size distributions of CDs (**A**) and GSH-AuNCs (**B**). Insets showed particle size distribution histogram statistically obtained (red bar) and the normal distribution curve (black line).

**Figure 2:**
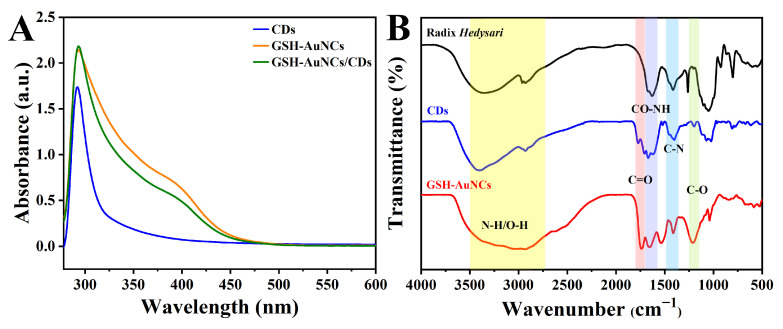
UV–vis absorbance spectra of CDs, GSH-AuNCs, and GSH-AuNCs/CDs nanohybrid (**A**). FT-IR spectra of Radix *Hedysari*, CDs, and GSH-AuNCs (**B**).

As shown in Scheme 1, CDs (Scheme 1A) and GSH-AuNCs (Scheme 1B) were synthesized in one simple step. GSH-AuNCs/CDs nanohybrid was prepared by simple physical mixing of CDs and GSH-AuNCs and the sensing principle of detecting CDZM in Scheme 1C. TEM was used to characterize the morphology and size of the GSH-AuNCs and CDs. The results revealed that both CDs and GSH-AuNCs were well-dispersed. As shown in Figure 1A,B, the particle size of CDs varied in a range of 0.86 nm–2.23 nm with average values of 1.45 nm. The particle size of GSH-AuNCs varied from 0.98 to 3.52 nm with average values of 1.88 nm. Figure 2A depicted the UV–vis absorption spectra of the CDs, GSH-AuNCs, and GSH-AuNCs/CDs nanohybrid. The CDs, GSH-AuNCs, and GSH-AuNCs/CDs nanohybrid all showed a strong UV absorption peak at 294 nm, which may be assigned to the n–π* transition. GSH-AuNCs and the GSH-AuNCs/CDs nanohybrid exhibited a slight shoulder around 390 nm. The FT-IR analysis of Radix *Hedysari*, CDs, and GSH-AuNCs was well-performed, with their unique peaks shown in Figure 2B. The absorption peaks of Radix *Hedysari* were mainly exhibited at 3363, 1629, and 1415 cm^−1^, corresponding to the absorption peaks of N−H, CO-NH, and C-N. The characteristic absorption peaks of CDs were mainly exhibited at 3402 and 1706 cm^−1^, corresponding to the absorption peaks of N−H and C=O [35], confirming the successful preparation of CDs. The absorption peaks of GSH-AuNCs were characterized at 3215 cm^−1^, 1737 cm^−1^, 1413 cm^−1^, and 1211 cm^−1^, corresponding to the stretching vibrations of the O–H, C=O, C–N, and C–O bonds [34], respectively. The XRD pattern of CDs (Figure S1) showed a wide peak around 21.4° corresponding to the amorphous carbon phase, with no crystalline phase in its structure.

**Figure 3:**
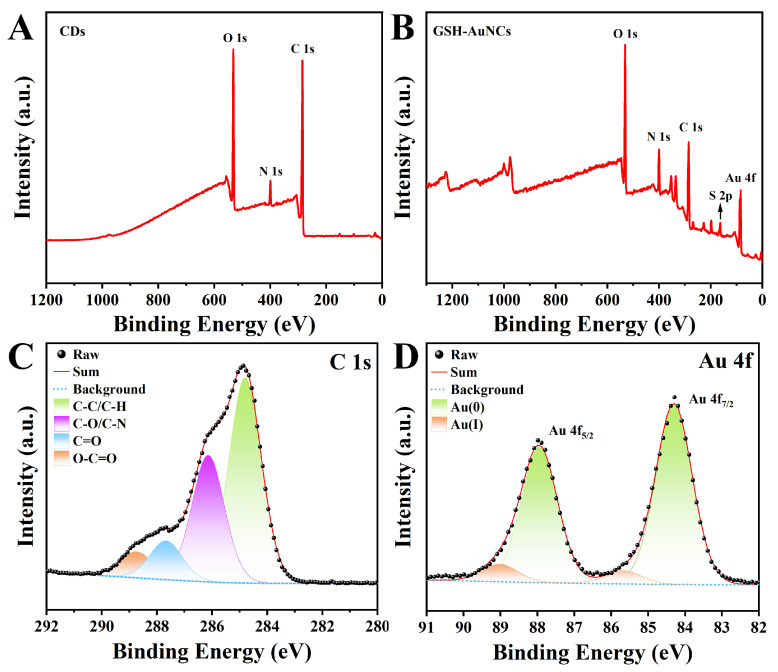
Full-scan survey XPS spectrum of CDs (**A**) and GSH-AuNCs (**B**). C 1s high-resolution XPS spectra of CDs. (**C**). Au 4f high-resolution XPS spectra of GSH-AuNCs (**D**).

Furthermore, the XPS of CDs and GSH-AuNCs were also characterized. The survey spectrum in Figure 3A,B confirmed the existence of C, N, and O elements in CDs, as well as the existence of Au, S, C, N, and O elements in GSH-AuNCs. The C 1s high-resolution XPS spectra of CDs (Figure 3C) displayed four characteristic peaks at around 284.76, 286.16, 287.66, and 288.76 which were associated with C-C/C-H, C-O/C-N, C=O, and O-C=O. The Au 4f region spectrum of GSH-AuNCs (Figure 3D) could be divided into two obvious peaks ascribed to Au 4f_7/2_ and Au 4f_5/2_, corresponding to the Au (0) (84.28 and 87.98 eV) core and the Au (I) (85.68 and 88.98 eV) shell.

### 2.2. Optical Properties of CDs, GSH-AuNCs, and GSH-AuNCs/CDs Nanohybrid

**Figure 4:**
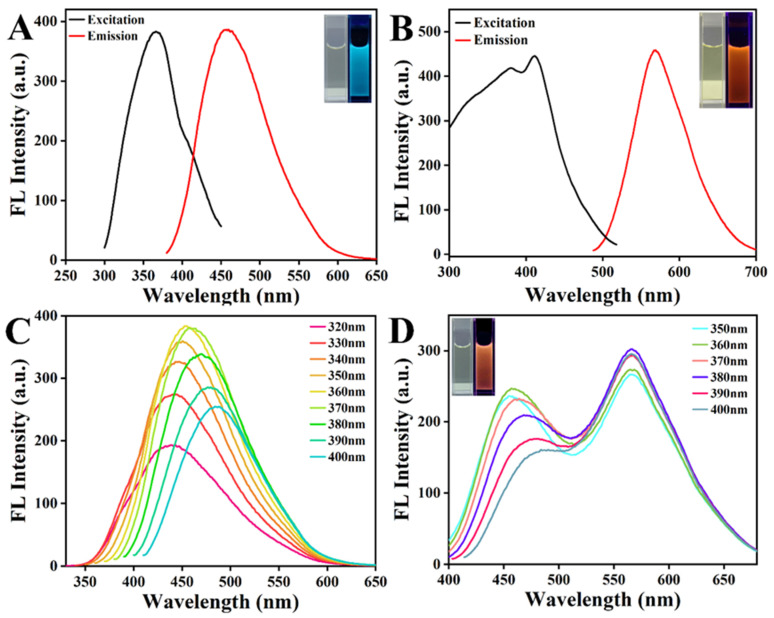
Fluorescence excitation spectra (Em = 458 nm) and emission spectra (Ex = 360 nm) of CDs (**A**) and fluorescence excitation spectra (Em = 569 nm) and emission spectra (Ex = 410 nm) of GSH-AuNCs (**B**). Fluorescence emission spectra of CDs at different excitation wavelengths (Ex = 320–400 nm) (**C**). Fluorescence emission spectra of GSH-AuNCs/CDs nanohybrid at different excitation wavelengths (Ex = 350–400 nm) (**D**). The insets showed the photographs of CDs, GSH-AuNCs, and GSH-AuNCs/CDs nanohybrid under daylight and 365 nm UV light, respectively.

As could be observed in Figure 4A,B, the maximum emission peak of CDs appeared at 458 nm when excited at 360 nm and that of GSH-AuNCs appeared at 569 nm when excited at 410 nm. Photographs showed that the CDs were colorless under daylight and displayed blue-light-emitting under 365 nm UV light. The GSH-AuNCs displayed a brown color in daylight while they displayed bright orange fluorescence under 365 nm UV light. Figure 4C,D exhibited the various fluorescence emission spectra of CDs and the GSH-AuNCs/CDs nanohybrid excited at a series of wavelengths. When the excitation wavelength varied from 320 to 400 nm, the maximum emission wavelength of CDs changed from 438 nm to 486 nm. This phenomenon confirmed the typical excitation-dependent fluorescence behavior of CDs, which was consistent with previously reported biomass-based carbon spots. This was mainly due to the existence of different particle sizes, diverse surface defect states, and multiple functional groups of CDs [36,37]. The fluorescence quantum yield of CDs was determined to be 2.35% by a transient steady-state fluorescence spectrometer. When the excitation wavelength varied from 350 to 400 nm, the maximum emission wavelength of CDs in the GSH-AuNCs/CDs nanohybrid changed from 456 nm to 483 nm while the maximum emission wavelength of GSH-AuNCs in the GSH-AuNCs/CDs nanohybrid had not changed obviously. When the excitation wavelength was 360 nm, the intensity and wavelength of the GSH-AuNCs/CDs nanohybrid were most suitable. Therefore, 360 nm was chosen as the excitation wavelength in subsequent experiments. As shown, the GSH-AuNCs/CDs nanohybrid was colorless under daylight, while they emitted orange fluorescence under 365 nm UV light.

Figure S2 showed that, after the physical mixing of CDs and GSH-AuNCs, the fluorescence intensity of CDs decreased while that of GSH-AuNCs was almost unchanged. It can be seen from Figure 2A and 4A that the fluorescence spectra of the CDs were effectively overlapped by the UV–vis absorption spectrum of GSH-AuNCs at 400–450 nm. In addition, no new absorption peak appeared in the UV–vis absorbance spectrum of the GSH-AuNCs/CDs nanohybrid, indicating that no new complexes were formed. Therefore, the mechanism may be fluorescence resonance energy transfer (FRET) or inner filter effect (IFE). Moreover, the results suggested the Zeta potentials of the CDs, GSH-AuNCs, and GSH-AuNCs/CDs nanohybrid were 17.6, 17.8, and 7.4 mV, respectively (Figure S3), all of which were positively charged, indicating the existence of electrostatic repulsion between CDs and GSH-AuNCs. Therefore, CDs and GSH-AuNCs may not be close to generate FRET. In conclusion, the possible mechanism could be primarily owing to IFE.

### 2.3. Fluorescence Stability of GSH-AuNCs/CDs Nanohybrid

**Figure 5:**
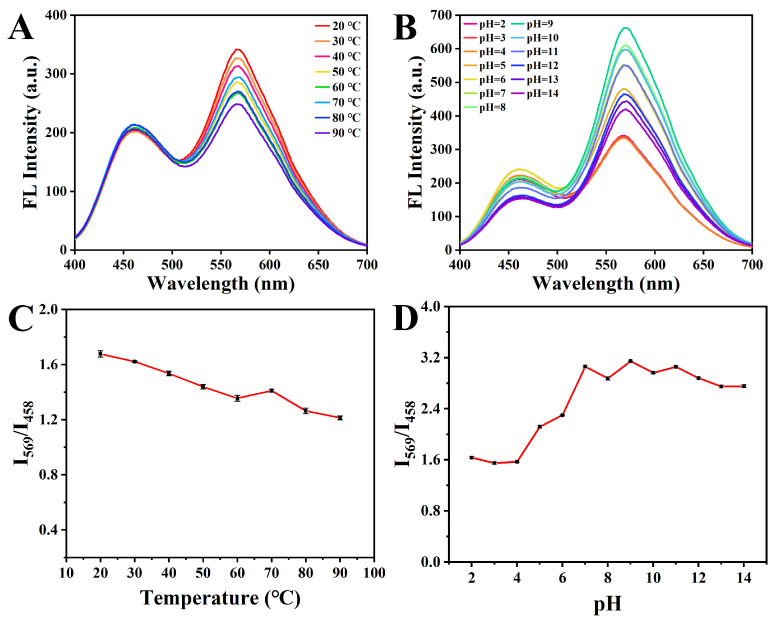
Ratio-fluorescence spectra of GSH-AuNCs/CDs nanohybrid at temperature (**A**) ranging from 20 to 90 °C and pH (**B**) ranging from 2 to 14. The effects of temperature (**C**) and pH (**D**) on fluorescence of GSH-AuNCs/CDs nanohybrid.

It is of great significance that we investigate the stability of the fluorescent probe for practical application. Thus, external influences such as temperature and pH were discussed in detailed. First, the influence of temperature was tested in the range of 20–90 °C. As shown in Figure 5A,C, the fluorescence intensity ratio gradually decreased with the increase in temperature. The fluorescence intensity ratio slightly decreased with the increase in temperature. In general, the I_569_/I_458_ fluorescence intensity ratio did not change significantly. Then, the influence of pH was investigated in the range of 2–14. When pH was 2–4, the I_569_/I_458_ fluorescence intensity ratio did not change obviously. However, the intensity of ratio fluorescence was significantly enhanced when the pH ranged from 5 to 14. It showed that, when the temperature of the nanohybrid was restored from 90 °C to room temperature, the fluorescence intensity recovered (Figure S4). However, when the pH was adjusted from 14 to 2, the fluorescence intensity did not fully recover (Figure S5). According to the above phenomena, 20 °C and pH 2 were selected as the optimal conditions for fluorescent probes.

### 2.4. Ratio-Fluorescence Detection of CDZM

According to the above stability investigation, a series concentration of CDZM was added into the GSH-AuNCs/CDs nanohybrid at room temperature with the pH of 2. The addition of CDZM would slightly quench the fluorescence of CDs, whereas the fluorescence of GSH-AuNCs was obviously enhanced. The sensing mechanism of CDZM was suspected as follows: when CDZM was added to the GSH-AuNCs/CDs nanohybrid, functional groups such as carboxyl and amine groups on the CDZM form strong bonds with functional groups such as carboxyl, amine, and carbonyl groups on GSH-AuNCs through chemical bonding, while there was no obvious interaction between CDZM and CDs. CDs acted as reference signals that also played an important role in ratio fluorescence. The addition of CDZM triggered the aggregation of GSH-AuNCs, leading to the aggregation-induced emission effect (AIE). As a result, GSH-AuNCs enhanced fluorescence at 569 nm while CDs showed little change at 458 nm, achieving the purpose of CDZM detection.

**Figure 6:**
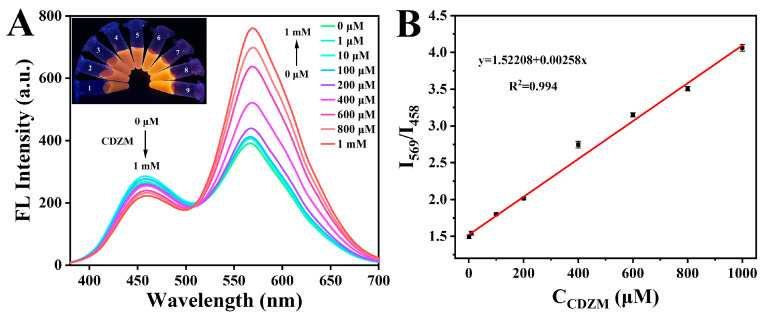
Ratio-fluorescence spectra of various concentrations of CDZM from 1 to 1000 μM (**A**). The linear of I_569_/I_458_ with different concentrations of CDZM (1–1000 μM) (**B**). The insets showed the photographs of GSH-AuNCs/CDs nanohybrid with the addition of various concentrations of CDZM under daylight and 365 nm UV light (the numbers 1 to 9 were 0, 1, 10, 100, 200, 400, 600, and 800 μM, and 1 mM respectively).

Based on this, the analytical values for CDZM detection were further explored, including the sensitivity, accuracy, selectivity, and applicability. With the increase in CDZM concentrations ranging from 1 to 1000 μM, the blue fluorescence of the CDs at 458 nm gradually declined, whereas the orange fluorescence of GSH-AuNCs increased at 569 nm when excited by 360 nm (Figure 6A). The result was shown in Figure 6B; a good linear relationship between I_569_/I_458_ and the concentration of CDZM was well-established: I_569_/I_458_ = 1.52208 + 0.00258x (R^2^ = 0.994). The limit of detection (LOD) was calculated to be 0.19 μM, according to the formula LOD = 3 σ/s, where σ represented the blank standard deviation (n = 10) and s was the slope of the linear regression equation.

### 2.5. Selectivity Study

**Figure 7:**
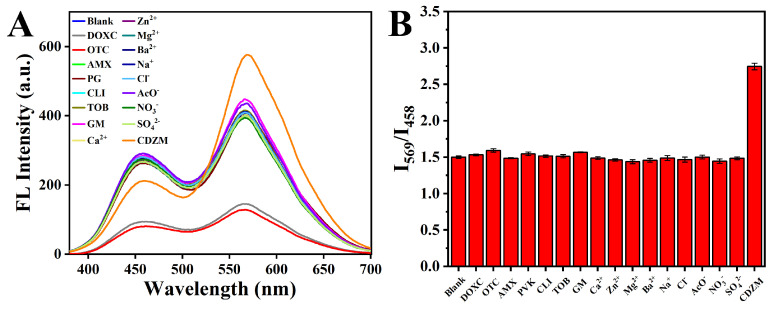
Fluorescence spectra of the fluorescent probe with the addition of the interferents and CDZM (**A**). The fluorescence intensity ratio after adding the interferents and CDZM (**B**).

In addition to sensitivity, selectivity is also an important factor in evaluating the detection effect of fluorescent probes. To evaluate the selectivity of the GSH-AuNCs/CDs nanohybrid for CDZM, the effect of typical interfering substances, such as doxycycline (DOXC), oxytetracycline (OTC), amoxicillin (AMX), benzylpenicillin potassium (PVK), clindamycin (CLI), tobramycin (TOB), gentamicin (GM), metal ions (Ca^2+^, Zn^2+^, Mg^2+^, Ba^2+^, and Na^+^), and anions (Cl^−^, AcO^−^, NO_3_^−^, and SO_4_^2−^) was investigated. The above interferents were added into the GSH-AuNCs/CDs nanohybrid with the final concentration of 400 μM, with the results shown in Figure 7A,B. Obviously, all of the above interferents including metal ions and anions could not induce a significant change in the fluorescence spectra and fluorescence intensity ratio (I_569_/I_458_) of the probes. Therefore, the fluorescence intensity of DOXC and OTC decreased significantly compared with the blank sample, but the fluorescence intensity ratio did not change visibly, whereas CDZM was found to be capable of quenching the fluorescence of CDs while improving the fluorescence of GSH-AuNCs, suggesting that the nanohybrid exhibited a good selectivity for CDZM detection, providing a possibility to be applied in actual samples.

### 2.6. Determination of CDZM in Real Samples

**Figure 8:**
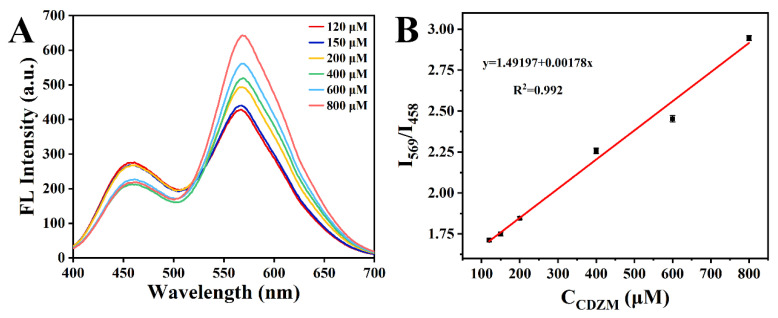
Ratio-fluorescence spectra of various concentrations of CDZM from 120 to 800 μM in urine samples (**A**). The linear of I_569_/I_458_ with different concentrations of CDZM in urine samples (120–800 μM) (**B**).

To verify the practical application of the GSH-AuNCs/CDs nanohybrid, urine samples were prepared for a feasibility study evaluation. The urine samples were simply pretreated according to Section 3.9, and then spiked with a series concentration of CDZM. The ratio-fluorescence spectra of various concentrations of CDZM from 120 to 800 μM in urine are shown in Figure 8A. A linear regression equation illustrated in Figure 8B was well-established between the I_569_/I_458_ fluorescence intensity ratio and CDZM concentration in the range of 120–800 μM as follows: I_569_/I_458_ = 1.49197 + 0.00178x (R^2^ = 0.992). The results showed that the method was reliable and suitable for the detection of CDZM in real samples.

The comparison of detection methods for CDZM and its analogues was summarized in Table S1. Since there were currently few methods for detecting CDZM, especially in fluorescence sensing, we also listed its analogues for comparison. The sensing detection method for CDZM was first reported in our article.

## 3. Materials and Methods 

### 3.1. Materials

HAuCl_4_·3H_2_O was purchased from Sigma-Aldrich (Shanghai, China). The Radix *Hedysari* was purchased at local medicinal markets. L-glutathione (GSH) was purchased from Shanghai Aladdin Biochemical Technology Co., Ltd. (Shanghai, China). Benzylpenicillin potassium (PVK) and gentamicin (GM) were purchased from Shanghai Yuanye Bio-Technology Co., Ltd. (Shanghai, China). Tobramycin (TOB), oxytetracycline (OTC), doxycycline (DOXC), clindamycin (CLI), amoxicillin (AMX), and cefodizime sodium (CDZM) were purchased from Macklin Biochemical Co., Ltd. (Shanghai, China). NaCl, MgCl_2_·6H_2_O, and Na_2_SO_4_ were purchased from Da Mao Chemical Reagent Factory (Tianjin, China). Zn(NO_3_)_2_·6H_2_O, CaCl_2_, and BaCl_2_·2H_2_O were purchased from Beijing Hong Xing Chemical Plant (Beijing, China). CH_3_COONa was purchased from Beijing Shuang Huan Chemical Reagent Factory (Beijing, China). All the chemical reagents were of analytical grade and used without further purification. Double-deionized water (DDW, A.S. Watson, Beijing, China) was used throughout the analysis.

### 3.2. Characterizations

The fluorescent spectra were recorded by Spectro fluorophotometer (Shimadzu, RF-5301 PC, Kyoto, Japan). The slit width of excitation and emission light was 5 nm. Fourier-transform infrared studies (FT-IR) spectra were conducted using an FT-IR spectrometer (Nicolet, Nexus 870, Green Bay, WI, USA). The surface composition of the samples was analyzed by X-ray Photoelectron Spectrometer (XPS) (Shimadzu, Axis Supra, Kyoto, Japan). Transmission electron microscopy (TEM) images were obtained using an FEI Tecnai G2TF20 (Feitecnai, Hillsboro, OR, USA) transmission electron microscope. Ultraviolet absorption spectra were recorded by ultraviolet–visible (UV–vis) spectroscopy (Perkin Elmer, lamda25, Waltham, MA, USA). X-ray diffraction (XRD) patterns were given by X-ray diffractometer (Rigaku, D/MAX 2550, Tokyo Japan). Zeta potentials were measured by dynamic laser light scattering (Malvern, ZEN3690, Malvern, Britain).

### 3.3. Preparation of GSH-AuNCs

The GSH-AuNCs were prepared according to previously reported study with minor modifications [38]. Briefly, HAuCl_4_·3H_2_O solution (4.0 mL, 20 mM) was heated to 70 °C for 5 min. The GSH solution (36 mL, 3.3 mM) was quickly added to the vigorously agitated HAuCl_4_·3H_2_O solution and the mixture was incubated at 70 °C for 24 h with constant stirring. It was observed that the color of the solution changed from colorless to yellow. Finally, the GSH-AuNCs were obtained and stored at 4 °C for use.

### 3.4. Preparation of CDs

The CDs were synthesized via a simple one-step hydrothermal route [16]. In brief, 0.2 g Radix *Hedysari* powder and 15 mL of water were mixed, and we sonicated the mixture for about 40 min. Then, the solution was placed in a 50 mL Teflon-lined, stainless-steel autoclave. Afterward, the autoclave was maintained at a temperature of 190 °C for 5 h for reaction. After the solution had cooled to room temperature, the mixture was filtered through a 0.22 μm microporous membrane. Eventually, the CDs was stored at 4 °C for further utilization.

### 3.5. Fabrication of GSH-AuNCs/CDs Nanohybrid

The ratio-fluorescence probe was obtained by mixing GSH-AuNCs and CDs in an appropriate intensity ratio. In a nutshell, 1.0 mL of CDs and 9.0 mL of GSH-AuNCs were mixed and vigorously stirred for 1 min, and the resulting GSH-AuNCs/CDs nanohybrid solution was kept at 4 °C for further utilization.

### 3.6. Stability of GSH-AuNCs/CDs Nanohybrid

To investigate the influence of pH and temperature on GSH-AuNCs/CDs nanohybrid fluorescence intensity, the solution pH was adjusted from 2 to 14 by using HCl and NaOH at a concentration of 0.5 M, respectively, and the temperature in the range of 20–90 °C.

### 3.7. Fluorescence Detection of CDZM

The fluorescence detection of CDZM was performed at room temperature as follows. In a typical experimental procedure, 360 nm was chosen as the optimum excitation wavelength and the emission wavelength was in the range of 370–700 nm. First, 450 μL of the GSH-AuNCs/CDs nanohybrid was injected into a centrifuge tube. The fluorescence signal of the GSH-AuNCs/CDs nanohybrid at 569 nm and 458 nm (λ_ex_ = 360 nm) were measured. Then, 50 μL of CDZM with different concentrations (1, 10, 100, 200, 400, 600, 800, and 1000 μM) were added to the GSH-AuNCs/CDs nanohybrid solutions. Then, the mixture was kept at 20 °C for 5 min. Fluorescence intensity ratio (I_569_/I_458_) was calculated as the response signal.

### 3.8. Selectivity Study

The selectivity of the GSH-AuNCs/CDs nanohybrid towards CDZM was examined by adding representative interference agents including DOXC, OTC, AMX, PVK, CLI, TOB, GM, metal ions (Ca^2+^, Zn^2+^, Mg^2+^, Ba^2+^, and Na^+^), and anions (Cl^−^, AcO^−^, NO_3_^−^, and SO_4_^2−^). Additionally, the fluorescence intensity of the GSH-AuNCs/CDs nanohybrid was also recorded. The ultimate concentration of interferents was 400 μM.

### 3.9. Real-Sample Analysis

Urine samples were pretreated according to the usual procedure [39]. Briefly, samples were centrifuged at 12,000 rpm for 10 min and the supernatant was filtered through a 0.22 μm membrane. The supernatant was diluted 200-fold and placed in a 4 °C refrigerator. Finally, a series of CDZM solutions at various concentrations were prepared by treated urine sample solution and the following detection steps were shown in Section 3.7.

## 4. Conclusions

In this work, we effectively constructed a dual-emitting GSH-AuNCs/CDs nanohybrid fluorescent probe, exhibiting the significant blue and orange fluorescence emission. The GSH-AuNCs/CDs nanohybrid showed an excellent fluorescence sensing ability for CDZM with the analytical values of high sensitivity, good accuracy, and approved selectivity. Moreover, the GSH-AuNCs/CDs nanohybrid sensor was successfully applied in urine sample detection. It served to show that dual-band ratio fluorescence had advantages in the detection of complex biological samples, which provided a great potential for future market applications.

## Data Availability

The data are available upon request due to restrictions, e.g., privacy or ethical. The data presented in this study are available upon request from the corresponding author.

## Supplementary Materials

The following supporting information can be downloaded at: https://www.mdpi.com/article/10.3390/ijms25115971/s1. References [40,41,42,43,44] are cited in the supplementary materials.
