# Supplementary material for: Design of Ratio-Fluorescence Nanohybrid Based on Radix Hedysari Green-Synthesized CDs and GSH-AuNCs for Sensitive Detection of Cefodizime Sodium in Urine Sample
Source: Int J Mol Sci. 2024 May 29;25(11):5971. doi: 10.3390/ijms25115971 (PMC11172498; doi:10.3390/ijms25115971)
Supplement: Supplementary file 1 [file ijms-25-05971-s001.zip › ijms-3006114-supplementary.pdf]

Supporting Information

Design of Ratio-Fluorescence Nanohybrid Based on Radix *Hedysari* Green-Synthesized CDs and GSH-AuNCs for Sensitive Detection of Cefodizime Sodium in Urine Sample

Yan-Xin Guo, Xin-Ran Guo and Xin-Yue Chen *

Institute of Pharmaceutical Analysis, School of Pharmacy, Lanzhou University, Lanzhou 730030, China; gyanxin2023@lzu.edu.cn (Y.-X.G.); guoxr21@lzu.edu.cn (X.-R.G.)

* Correspondence: cxy@lzu.edu.cn or chenxinyue888@126.com

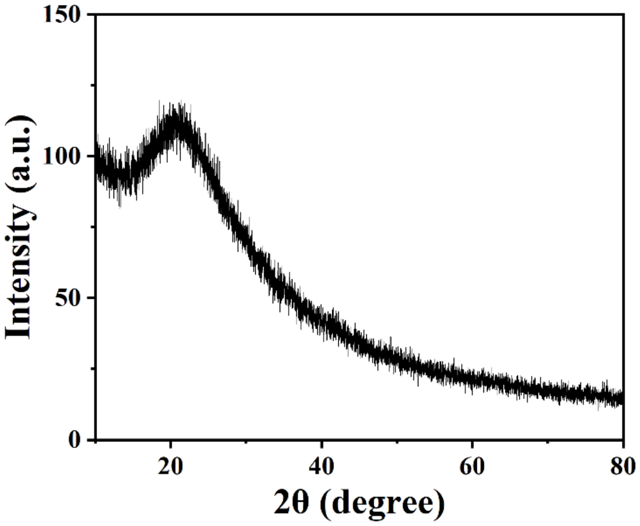

Figure S1. XRD pattern of CDs

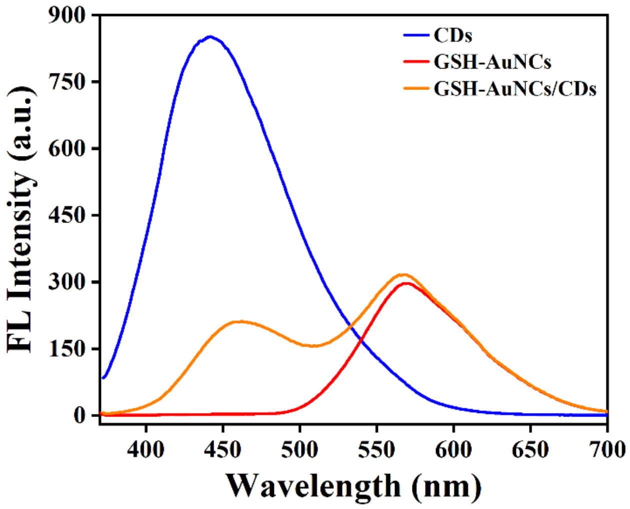

Figure S2. Fluorescence spectra of CDs, GSH-AuNCs and GSH-AuNCs/CDs nanohybrid.

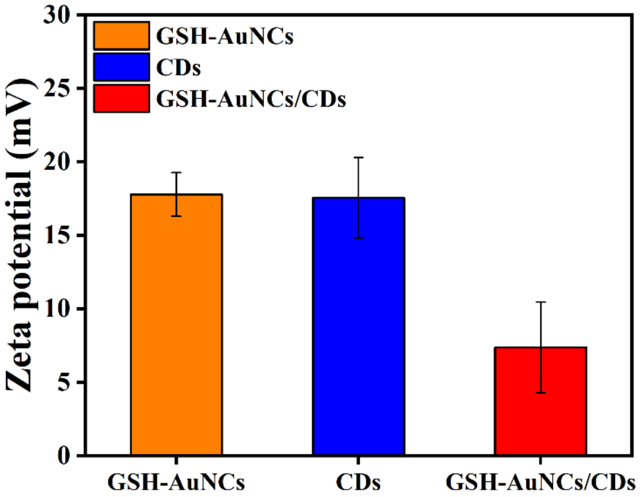

Figure S3. The corresponding Zeta potentials of CDs, AuNCs and GSH-AuNCs/CDs nanohybrid.

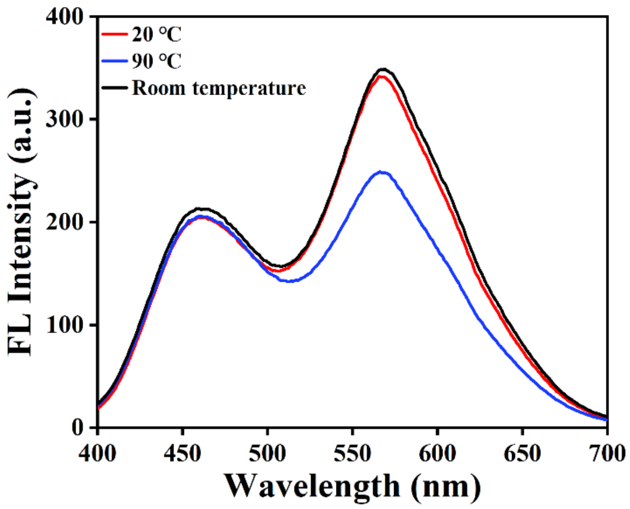

Figure S4. Ratio fluorescence spectra of GSH-AuNCs/CDs nanohybrid at temperature 20°C, 90°C and back to room temperature.

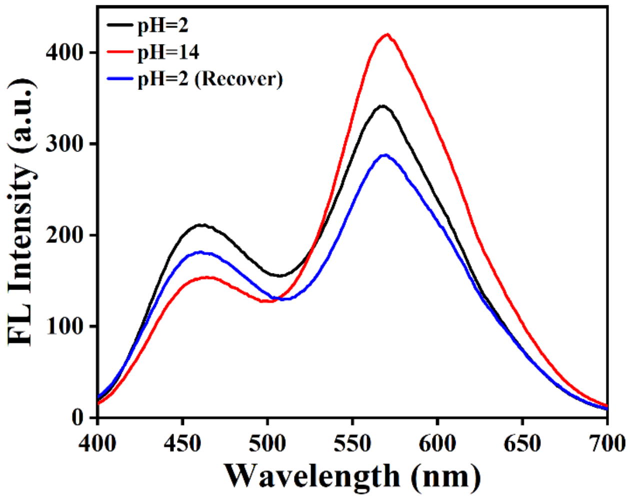

Figure S5. Ratio fluorescence spectra of GSH-AuNCs/CDs nanohybrid at pH 2, 14 and back to 2.

Table S1

Comparison of different detection methods of CDZM and its analogues.

Detection object	Method	Linear range	LOD	Reference
Ceftriaxone sodium (CTR)	I-CDs	0–30 μM	19.7 nM	[1]
Cephalexin (CFX)	gCDs/AuNCs@ZIF-8	0.1–6 ng/mL	0.04 ng/mL	[2]
Cephalexin (CFX)	CDs@SiO_2-r-QDs	1–500 μM	0.7 μM	[3]
CDZM	HPLC	1–400 μg/mL	0.1 μg/mL	[4]
CDZM	UV detection and HPLC	2–250 μg/mL	0.5 μg/mL	[5]
CDZM	GSH-AuNCs/CDs	1-1000 μM	0.19 μM	This work

References

1. Zhang, Q.; Wang, L.; Su, P.; Yu, L.; Yin, R.; Bu, Y.; Hao, X.; Sun, M.; Wang, S. Highly selective and sensitive determination of ceftriaxone sodium using nitrogen-rich carbon dots based on ratiometric fluorescence. *Talanta* **2023**, *255*, 124205, doi:https://doi.org/10.1016/j.talanta.2022.124205.
2. Jalili, R.; Irani-nezhad, M.H.; Khataee, A.; Joo, S.W. A ratiometric fluorescent probe based on carbon dots and gold nanocluster encapsulated metal–organic framework for detection of cephalexin residues in milk. *Spectrochimica Acta Part A: Molecular and Biomolecular Spectroscopy* **2021**, *262*, 120089, doi:https://doi.org/10.1016/j.saa.2021.120089.
3. Hao, A.-Y.; Wang, X.-Q.; Mei, Y.-Z.; Nie, J.-F.; Yang, Y.-Q.; Dai, C.-C. A smartphone-combined ratiometric fluorescence probe for specifically and visibly detecting cephalexin. *Spectrochimica Acta Part A: Molecular and Biomolecular Spectroscopy* **2021**, *249*, 119310, doi:https://doi.org/10.1016/j.saa.2020.119310.
4. Bompadre, S.; Ferrante, L.; Leone, L.; De Martinis, M.; Ginaldi, L.; Quaglino, D. Determination of Cefodizime in Human Plasma by High-Performance Liquid Chromatography with Column-Switching. *Journal of Liquid Chromatography* **1995**, *18*, 2895-2909, doi:10.1080/10826079508009333.
5. Bompadre, S.; Ferrante, L.; Leone, L. On-line solid-phase extraction of cephalosporins. *Journal of Chromatography A* **1998**, *812*, 191-196, doi:https://doi.org/10.1016/S0021-9673(98)00382-3.
